# Crystal structures of sulfonamide protected bicyclic guanidines: (*S*)-8-{[(*tert*-butyl­dimethyl­sil­yl)­oxy]meth­yl}-1-[(2,2,4,6,7-penta­methyl-2,3-di­hydro­benzo­furan-5-yl)sulfon­yl]-1,3,4,6,7,8-hexa­hydro-2*H*-pyrimido[1,2-*a*]pyrimidin-1-ium tri­fluoro­methane­sulfonate and (*S*)-8-(iodo­meth­yl)-1-tosyl-1,3,4,6,7,8-hexa­hydro-2*H*-pyrimido[1,2-*a*]pyrimidin-1-ium iodide

**DOI:** 10.1107/S2056989024001129

**Published:** 2024-02-20

**Authors:** Jamal M. H. Alaboosh, Steven P. Hill, Benson M. Kariuki, James E. Redman

**Affiliations:** a School of Chemistry, Cardiff University, Main Building, Park Place, Cardiff, CF10 3AT, United Kingdom; Universidad de Los Andes Mérida, Venezuela

**Keywords:** crystal structure, bicyclic guanidine, Pbf, tos­yl

## Abstract

Two compounds, (*S*)-8-{[(*tert*-butyl­dimethyl­sil­yl)­oxy]meth­yl}-1-[(2,2,4,6,7-penta­methyl-2,3-di­hydro­benzo­furan-5-yl)sulfon­yl]-1,3,4,6,7,8-hexa­hydro-2*H*-pyrimido[1,2-*a*]pyrimidin-1-ium tri­fluoro­methane­sulfonate, C_27_H_46_N_3_O_4_SSi^+^·CF_3_O_3_S^−^, (**1**) and (*S*)-8-(iodo­meth­yl)-1-tosyl-1,3,4,6,7,8-hexa­hydro-2*H*-pyrimido[1,2-*a*]pyrimidin-1-ium iodide, C_15_H_21_IN_3_O_2_S^+^·I^−^, (**2**), have been synthesized and characterized. They are bicyclic guanidinium salts and were synthesized from *N*-(*tert*-but­oxy­carbon­yl)-l-me­thio­nine (Boc-l-Met-OH). The guanidine is protected by a 2,2,4,6,7-penta­methyl­dihydro­benzo­furan-5-sulfonyl (Pbf, **1**) or a tosyl (**2**) group. In the crystals of both compounds, the guanidinium group is almost planar and the N–H forms an intra­molecular hydrogen bond in a six-membered ring to the oxygen atom of the sulfonamide protecting group.

## Abstract 2

The crystal structures of two sulfonamide-protected bicyclic guanidine salts are reported. The structures feature intra­molecular N—H⋯O hydrogen bonding between the guanidinium group and the sulfonamide.

## 1. Chemical context

Cyclic guanidines have been observed as a structural motif in alkaloid natural products and been extensively explored as organocatalysts, ligands and receptors, among other applications (Chou *et al.*, 2019 ▸; Dong *et al.*, 2018 ▸; Lemrová & Soural, 2015 ▸; Selig, 2013 ▸; Fu & Tan, 2011 ▸; Coles, 2009 ▸; Leow & Tan, 2009 ▸; Best *et al.*, 2003 ▸). We have prepared sulfonamide-protected bicyclic guanidine derivatives **1** and **2** with a view towards incorporating the bicyclic guanidine moiety into synthetic peptides as torsionally constrained mimics of arginine. 
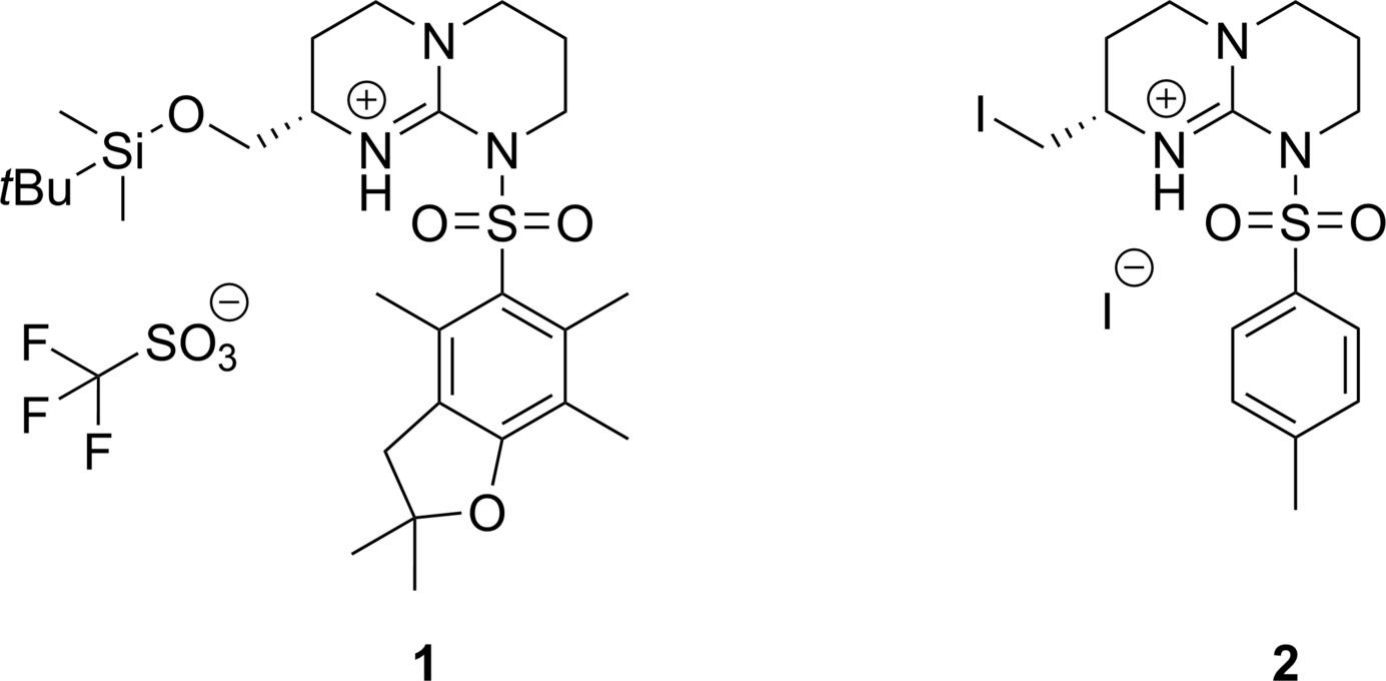


## 2. Structural commentary

**Figure 1:**
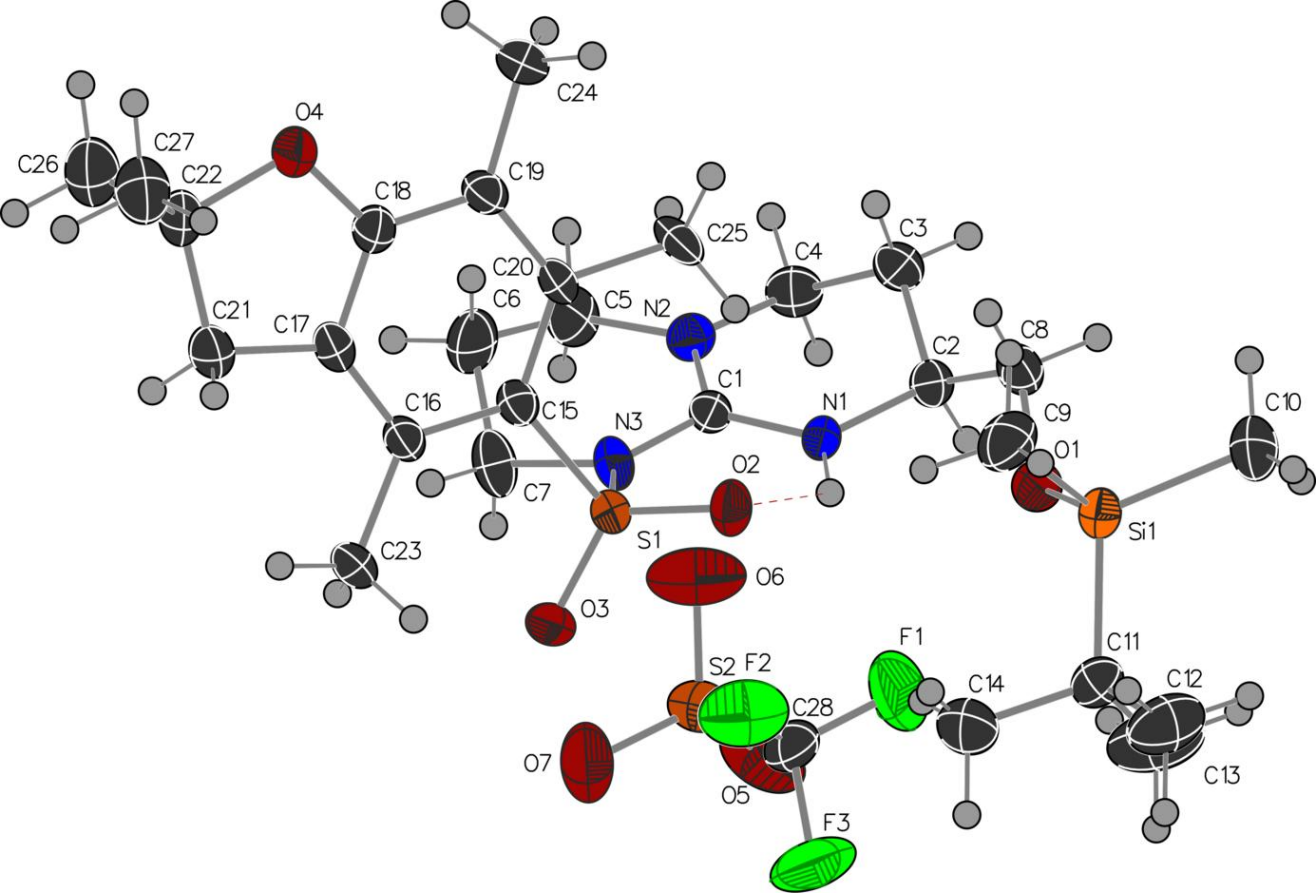
The mol­ecular structure of **1** with ellipsoids at the 50% probability level.

The mol­ecular structures of **1** and **2** are shown in Figs. 1 ▸ and 2 ▸, respectively. The guanidine group is protonated in both cases and the central carbon (C1) adopts an essentially planar geometry with N—C—N bond angles close to 120°. The C1—N1 bond lengths of 1.323 (4) and 1.328 (6) Å (in **1** and **2**, respectively) are slightly shorter than the C1—N3 bond lengths of 1.380 (4) and 1.400 (7) Å. The sulfonamide group adopts a conformation that allows the formation of an intra­molecular NH⋯OS hydrogen bond (Figs. 1 ▸ and 2 ▸, Tables 1 ▸ and 2 ▸). A second less optimal intra­molecular N—H⋯O contact to the oxygen of the sil­oxy group is observed in compound **1**. The alkyl substituent on the six-membered ring lies in an equatorial-like conformation where the chiral centre originates from the starting material (Boc-l-Met-OH) used in the synthesis.

## 3. Supra­molecular features

**Figure 3:**
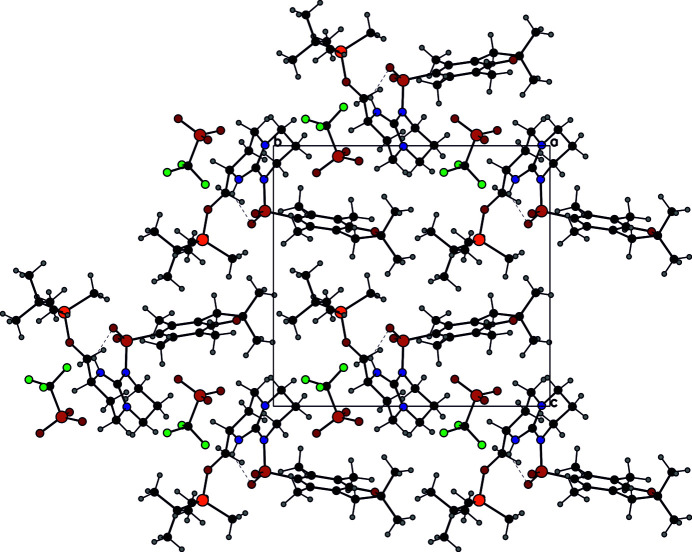
View of the packing of **1** along the *a* axis.

**Figure 4:**
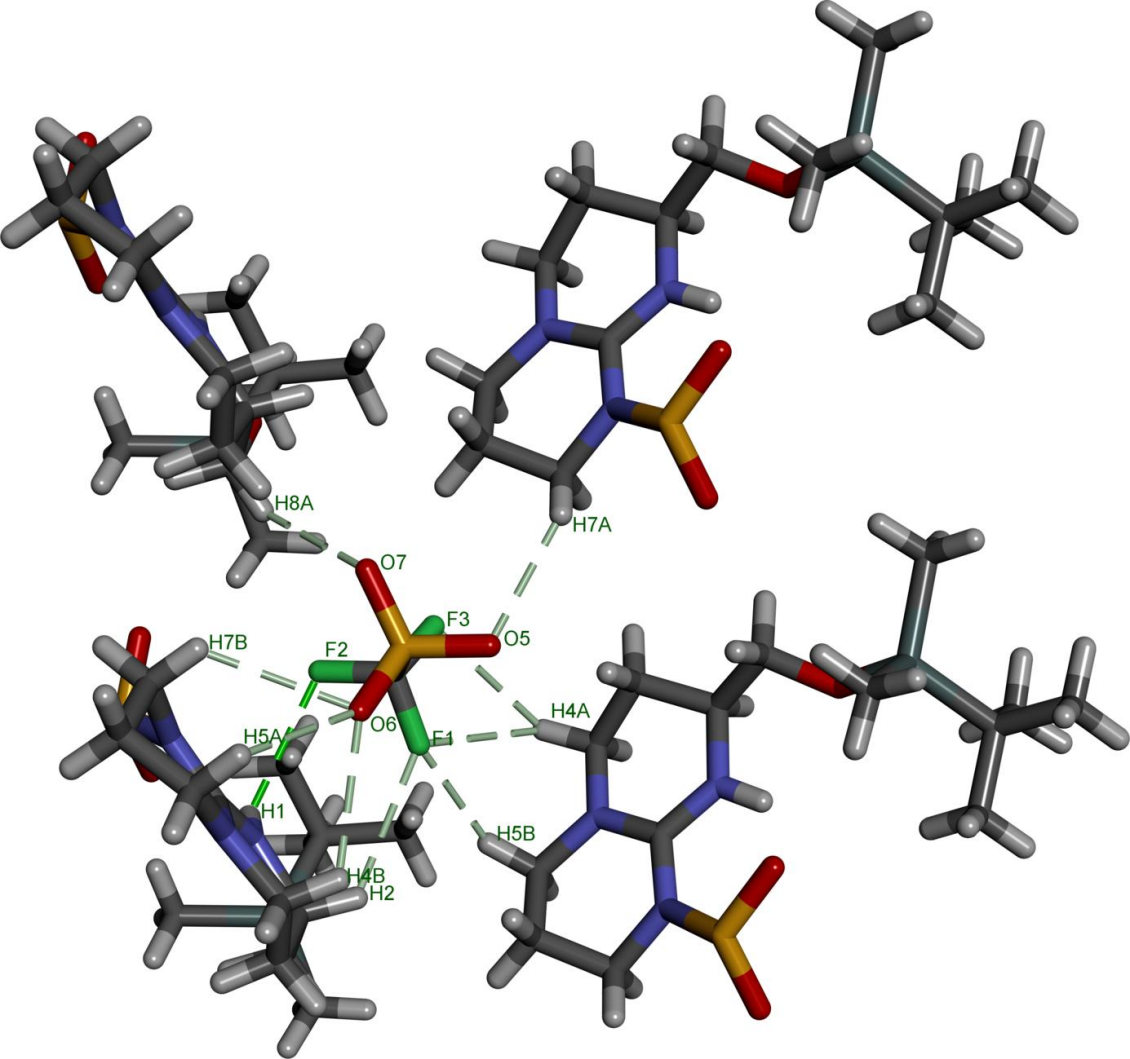
View of the packing of **1** around the tri­fluoro­methane­sulfonate anion. The penta­methyl-2,3-di­hydro­benzo­furan groups have been hidden for clarity.

Compound **1** packs with the guanidinium groups and triflate counter-ions arranged in layers perpendicular to the *c* axis (Fig. 3 ▸). These are inter­leaved with layers composed of the *tert*-butyl­dimethyl­silyl and 2,2,4,6,7-penta­methyl­dihydro­benzo­furan-5-sulfonyl (Pbf) protecting groups. Each triflate anion is surrounded by four cations, forming inter­actions with the C—H groups of the guanidinium as depicted in Fig. 4 ▸. Additionally, weak C–H⋯F and C—H⋯O inter­actions also consolidate the structure (Table 1 ▸).

**Figure 5:**
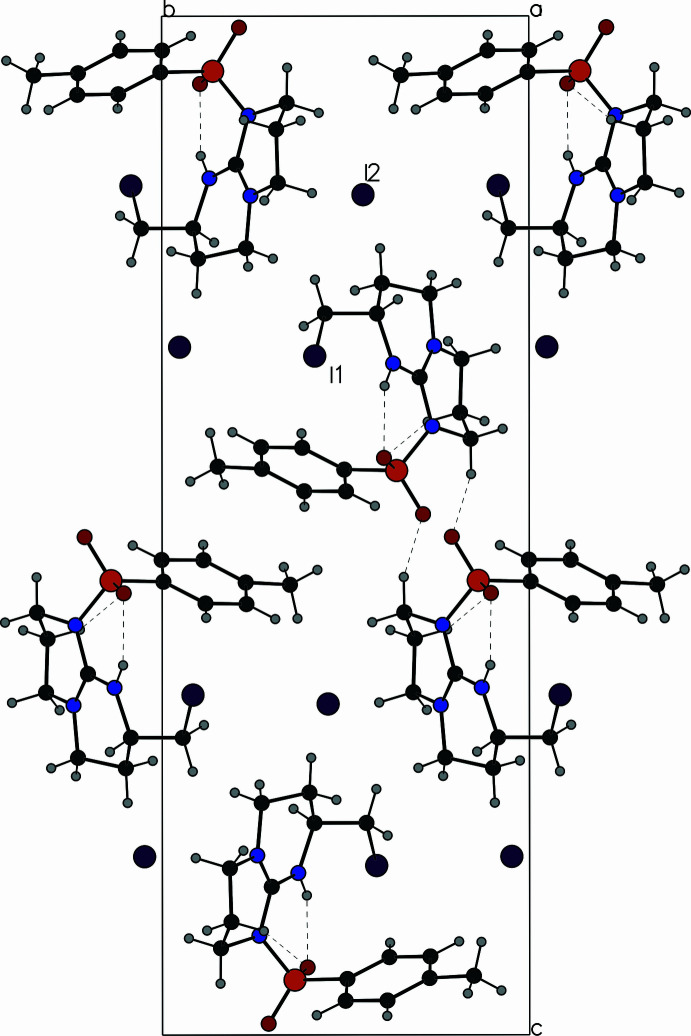
View of the packing of **2** along the *a* axis.

The mol­ecular packing of **2** is illustrated in Fig. 5 ▸. The tosyl groups of adjacent mol­ecules pack against each other placing the centroids of the aromatic rings 5.301 (4) Å apart. The methyl group forms a C—H⋯π inter­action such that the methyl carbon C15 lies 3.597 (9) Å from the centroid of the adjacent aromatic ring. The other face of the ring forms an inter­molecular inter­action with the iodine atom I1 that lies 3.600 (3) Å from the ring centroid. Weak C—H⋯O inter­actions also help to hold the structure together (Table 2 ▸).

## 4. Database survey

A search of the Cambridge Structural Database for sulfonyl guanidines revealed four related compounds. *N*,3-Diisopropyl-4-mesityl-1-[(4-methyl­phen­yl)sulfon­yl]imidazolidin-2-iminium tri­bromo­(methanol)zinc(II) methanol solvate (CSD refcode: FOFJIV; Craig II *et al.*, 2014 ▸) is a monocyclic guanidine bearing a tosyl group. This structure displays the same intra­molecular SO—HN hydrogen bond that is observed in **1** and **2**. 3,4,6,7,8,9-Hexa­hydro-2*H*-pyrimido[1,2-*a*]pyrimidin-1-ium-1-sulfinate (CSD refcode: SOWPOM; Adenot *et al.*, 2019 ▸) is a bicyclic guanidine–sulfur dioxide adduct that features a similar hydrogen bond. The guanidinium N to sulfonamide O distances in **1** and **2** are significantly shorter at 2.700 (4) and 2.737 (5) Å than the corresponding distances in related compounds that lack this intra­molecular inter­action. A neutral bicyclic tosyl­guanidine reported (CSD Refcode: WEWGAK) by Watanabe *et al.* (2023 ▸) places the sulfonamide oxygen 3.173 (5) Å from the guanidine nitro­gen. The hydrogen bond is also absent in *N*-(1,3-dibenzyl-1,3,4,4a,5,7a-hexa­hydro-2*H*-cyclo­penta­[*d*]pyrimidin-2-yl­idene)-2,2,5,7,8-penta­methyl­chro­mane-6-sulfonamide (CSD refcode: SIMSIS; Aranha Potter *et al.*, 2013 ▸) as the guanidinium group is fully alkyl­ated resulting in an N—O distance of 2.926 (3) Å.

## 5. Synthesis and crystallization

**Figure 6:**
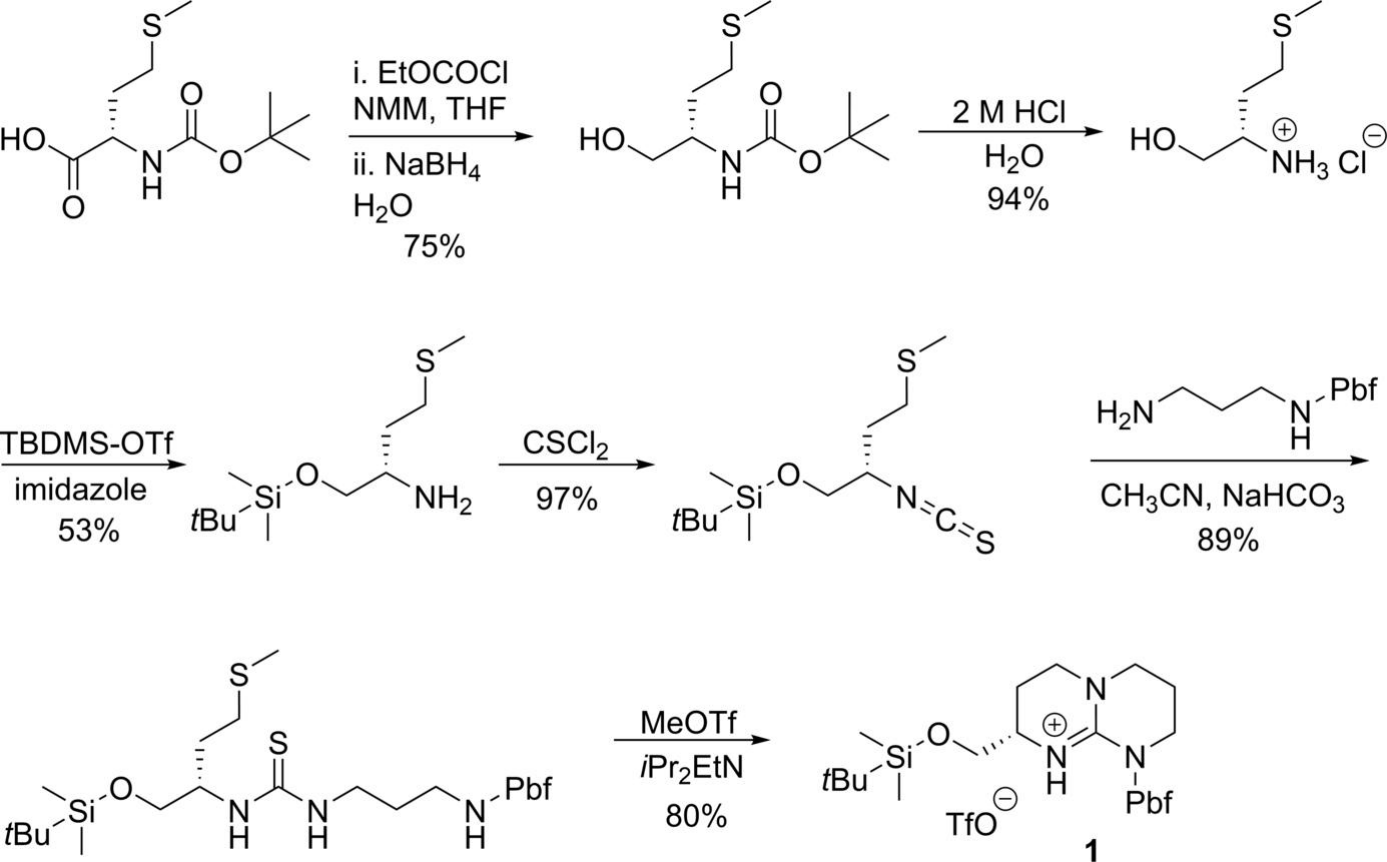
Synthetic scheme for **1**.

Compounds **1** and **2** were synthesized from Boc-l-Met-OH according to Figs. 6 ▸ and 7 ▸, respectively. Full synthetic procedures have been reported elsewhere (Alaboosh, 2017 ▸; Hill, 2012 ▸). Single crystals of **1** were grown by vapour diffusion from an EtOH/H_2_O solution. Single crystals of **2** were grown by vapour diffusion from a MeCN/Et_2_O solution.

Spectroscopic data for compound **1**:

**^1^H NMR (400 MHz, CDCl_3_) δ** 3.92–3.79 (*m*, 2H, SO_2_NCH_2_), 3.62–3.60 (*m*, 1H, OCHH), 3.33–3.28 (*m*, 1H, NCH), 3.12–2.99 (*m*, 4H, NCHCH_2_CH_2_, SO_2_NCH_2_CH_2_CH_2_), 2.83–2.80 (*m*, 1H, OCHH), 3.00 (*s*, 2H, furan-CH_2_), 2.52 (*s*, 3H, ArCH_3_), 2.48 (*s*, 3H, ArCH_3_), 2.13 (*s*, 3H, ArCH_3_), 2.06–1.93 (*m*, 3H, SO_2_NCH_2_CH_2_, NCHCH_2_), 1.46 [*s*, 6H, (CH_3_)_2_], 1.37–1.29 (*m*, 1H, NCHCHH), 0.85 (*s*, 9H, *t*-Bu), 0.01 [*s*, 6H, Si(CH_3_)_2_]. **^13^C NMR (100 MHz, CDCl_3_) δ** 159.1 (ArC), 143.2 (ArC), 137.7 (ArC), 132.7 (ArC), 124.3 (ArC), 117.1 (ArC), 86.4 (C(CH_3_)_2_), 60.3 (CH_2_), 50.3 (CH), 48.3 (CH_2_), 47.7 (CH_2_), 43.2 (CH_2_), 42.1 (CH_2_), 40.3 (CH_2_), 28.5 (CH_3_), 28.5 (CH_3_), 27.7 (CH_2_), 25.9 (*t*-Bu), 23.3 (CH_2_), 19.1 (CH_3_), 18.1 [C(CH_3_)_3_], 17.2 (CH_3_), 12.5 (CH_3_), −5.3 [Si(CH_3_)_2_]. **HRMS-ES+ (** ***m*** **/** ***z*** **):** calculated for C_27_H_46_N_3_O_4_SSi [*M* + H]^+^: 536.2973, found 536.2999.

Spectroscopic data for compound **2**:

**^1^H NMR (400 MHz, CD_3_OD) δ** 7.99 (*d*, 2H, ^3^ *J*_HH_ = 8.4 Hz, ArCH), 7.56 (*d*, 2H, ^3^ *J*_HH_ = 8.4 Hz, ArCH), 4.03–3.96 (*m*, 1H, SO_2_NCH*H*), 3.93–3.85 (*m*, 2H, SO_2_NCH*H*, NC*H*), 3.60–3.38 (*m*, 6H, ICH_2_, SO_2_NCH_2_CH_2_C*H*_2_, NCHCH_2_C*H*_2_), 2.49 (*s*, 3H, ArCH_3_), 2.24–2.17 (*m*, 1H, SO_2_NCH_2_C*H*_2_), 2.11–2.02 (*m*, 1H, SO_2_NCH_2_C*H*_2_), 1.92–1.83 (*m*, 1H, NCHC*H*_2_), 1.81–1.73 (*m*, 1H, NCHC*H*_2_). **^13^C NMR (125 MHz, CD_3_OD) δ** 150.9 (ArC), 148.7 (ArC), 135.0 (ArCH), 129.2 (ArCH), 52.7 (CH), 49.9 (CH_2_), 47.9 (CH_2_), 45.8 (CH_2_), 26.3 (CH_2_), 21.8 (CH_2_) 21.8 (CH_3_), 7.1 (CH_2_I). **HRMS-ESI+ (** ***m*** **/** ***z*** **):** calculated for C_15_H_21_IN_3_O_2_S [*M* + H]^+^: 434.0394, found: 434.0404.

## 6. Refinement

Crystallographic data for **1** were collected on an Agilent SuperNova Dual Atlas diffractometer with a mirror monochromator using Cu *K*α (λ = 1.5418 Å) radiation, equipped with an Oxford cryosystems cooling apparatus. Crystallographic data for **2** were collected at 150 K on a Nonius Kappa CCD diffractometer using graphite monochromated Mo *K*α radiation (λ = 0.71073 Å) equipped with an Oxford Cryosystems cooling apparatus.

Crystal data, data collection and structure refinement details are summarized in Table 3 ▸. The structures were solved using direct methods with *SHELXS* (Sheldrick, 2008 ▸) and refined with *SHELXL* (Sheldrick, 2015 ▸). All non-hydrogen atoms were refined anisotropically, while the hydrogen atoms were inserted in idealized positions with *U*_iso_ set at 1.2 or 1.5 times the *U*_eq_ of the parent atom. The absolute structures were determined based on the anomalous dispersion effects in the diffraction data. The Flack parameter for **1** indicated possible racemic twinning, which was confirmed by TWIN/BASF refinement to give *x* = 0.39. The value for **2** is consistent with an untwinned structure.

Images were produced using *Olex2-1.5* (Dolomanov *et al.*, 2009 ▸) and *Discovery Studio Visualizer* (v21.1.0.20298; BIOVIA, 2024 ▸). Hydrogen bonds and other inter­molecular contacts were identified with *PLATON* (Spek, 2020 ▸) and *Discovery Studio Visualizer*.

## Supplementary Material

Crystal structure: contains datablock(s) 1, 2, global. DOI: 10.1107/S2056989024001129/dj2073sup1.cif

Structure factors: contains datablock(s) 1. DOI: 10.1107/S2056989024001129/dj20731sup2.hkl

Supporting information file. DOI: 10.1107/S2056989024001129/dj20731sup4.cml

Structure factors: contains datablock(s) 2. DOI: 10.1107/S2056989024001129/dj20732sup3.hkl

Supporting information file. DOI: 10.1107/S2056989024001129/dj20732sup5.cml

CCDC references: 2330294, 2330293

Additional supporting information:  crystallographic information; 3D view; checkCIF report

## Figures and Tables

**Figure 2:**
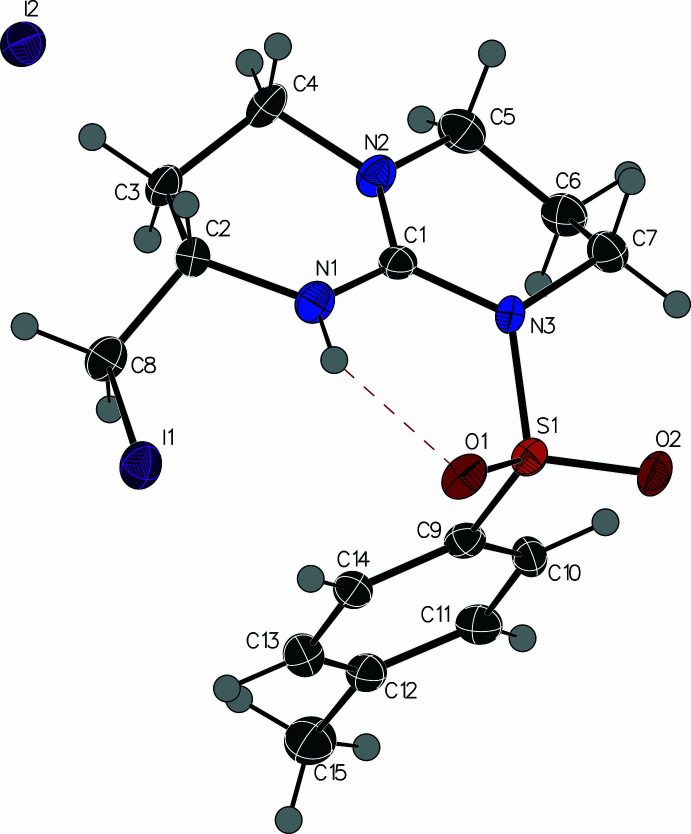
The mol­ecular structure of **2** with ellipsoids at the 50% probability level.

**Figure 7:**
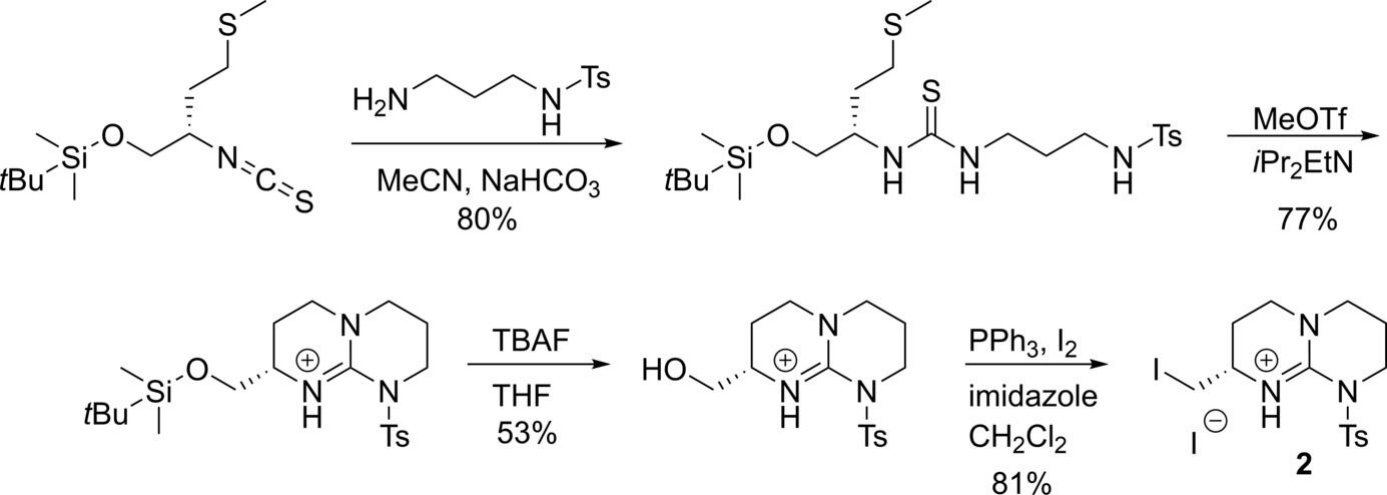
Synthetic scheme for **2**.

**Table 1:** Hydrogen-bond geometry (Å, °) for **1** [Chem scheme1]

*D*—H⋯*A*	*D*—H	H⋯*A*	*D*⋯*A*	*D*—H⋯*A*
N1—H1⋯O1	0.88	2.36	2.712 (4)	104
N1—H1⋯O2	0.88	2.07	2.700 (4)	127
C3—H3*B*⋯O7^i^	0.99	2.36	3.229 (5)	146
C5—H5*B*⋯F1^ii^	0.99	2.43	3.239 (6)	139
C24—H24*A*⋯O4	0.98	2.42	2.877 (4)	108
C25—H25*B*⋯O2	0.98	2.21	3.022 (5)	139
C7—H7*A*⋯O5^iii^	0.99	2.60	3.433 (5)	142
C24—H24*B*⋯O6^ii^	0.98	2.53	3.462 (5)	159

Symmetry codes: (i) 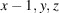 ; (ii) 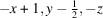 ; (iii) 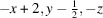 .

**Table 2:** Hydrogen-bond geometry (Å, °) for **2** [Chem scheme1]

*D*—H⋯*A*	*D*—H	H⋯*A*	*D*⋯*A*	*D*—H⋯*A*
N1—H1⋯I1	0.88	2.87	3.3359 (4)	115
N1—H1⋯O1	0.88	2.00	2.737 (5)	141
C7—H7*A*⋯O2	0.99	2.29	2.807 (8)	112
C14—H14⋯O1	0.95	2.57	2.934 (8)	103
C6—H6*B*⋯O1^i^	0.99	2.58	3.425 (8)	143
C7—H7*A*⋯O2^ii^	0.99	2.56	3.391 (8)	141
C10—H10⋯O2^ii^	0.95	2.49	3.441 (7)	174

Symmetry codes: (i) 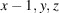 ; (ii) 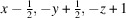 .

**Table 3:** Experimental details

	**1**	**2**
Crystal data		
Chemical formula	C_27_H_46_N_3_O_4_SSi^+^·CF_3_O_3_S^−^	C_15_H_21_IN_3_O_2_S^+^·I^−^
*M*_r_	685.89	561.21
Crystal system, space group	Monoclinic, *P*2_1_	Orthorhombic, *P*2_1_2_1_2_1_
Temperature (K)	150	150
*a*, *b*, *c* (Å)	8.5784 (3), 14.4797 (5), 13.6961 (5)	6.6117 (2), 10.1482 (2), 28.1444 (9)
α, β, γ (°)	90, 96.052 (4), 90	90, 90, 90
*V* (Å^3^)	1691.75 (10)	1888.40 (9)
*Z*	2	4
Radiation type	Cu *K*α	Mo *K*α
μ (mm^−1^)	2.32	3.45
Crystal size (mm)	0.33 × 0.06 × 0.04	0.41 × 0.20 × 0.20

Data collection		
Diffractometer	Agilent SuperNova, Dual, Cu at zero, Atlas	Nonius KappaCCD
Absorption correction	Gaussian (*CrysAlis PRO*; Agilent,2014 ▸)	Empirical (using intensity measurements) (*DENZO*/*SCALEPACK*; Otwinowski & Minor, 1997 ▸)
*T*_min_, *T*_max_	0.943, 0.988	0.339, 0.545
No. of measured, independent and observed [*I* > 2σ(*I*)] reflections	10136, 5593, 5350	4087, 4087, 3858
*R*_int_	0.027	0.030
(sin θ/λ)_max_ (Å^−1^)	0.623	0.648

Refinement		
*R*[*F* ^2^ > 2σ(*F* ^2^)], *wR*(*F* ^2^), *S*	0.037, 0.097, 1.02	0.031, 0.072, 1.04
No. of reflections	5593	4087
No. of parameters	408	207
No. of restraints	1	0
H-atom treatment	H-atom parameters constrained	H-atom parameters constrained
Δρ_max_, Δρ_min_ (e Å^−3^)	0.63, −0.48	0.77, −0.79
Absolute structure	Refined as an inversion twin	Flack *x* determined using 1475 quotients [(*I* ^+^)−(*I*[(*I* ^+^)−(*I* ^-^)]/[(*I* ^+^)+(*I* ^−^)] ^−^)]/[(*I* ^+^)+(*I* ^−^)] (Parsons et al., 2013 ▸)
Absolute structure parameter	0.39 (2)	−0.002 (19)

Computer programs: *CrysAlis PRO* (Agilent, 2014 ▸), *COLLECT* (Bruker, 2004 ▸), *HKL* *DENZO* and *SCALEPACK* (Otwinowski & Minor 1997 ▸), *SHELXS* (Sheldrick, 2008 ▸), *SHELXL2018/1* (Sheldrick, 2015 ▸), *OLEX2* (Dolomanov *et al.*, 2009 ▸) and *PLATON* (Spek, 2020 ▸).

## supplementary crystallographic information

### (S)-8-{[(tert-Butyldimethylsilyl)oxy]methyl}-1-[(2,2,4,6,7-pentamethyl-2,3-dihydrobenzofuran-5-yl)sulfonyl]-1,3,4,6,7,8-hexahydro-2H-pyrimido[1,2-a]pyrimidin-1-ium trifluoromethanesulfonate (1) Crystal data

**Table:**

C_27_H_46_N_3_O_4_SSi^+^·CF_3_O_3_S^−^	*F*(000) = 728
*M**_r_* = 685.89	*D*_x_ = 1.346 Mg m^−^^3^
Monoclinic, *P*2_1_	Cu *K*α radiation, λ = 1.54184 Å
*a* = 8.5784 (3) Å	Cell parameters from 3508 reflections
*b* = 14.4797 (5) Å	θ = 4.4–73.8°
*c* = 13.6961 (5) Å	µ = 2.32 mm^−^^1^
β = 96.052 (4)°	*T* = 150 K
*V* = 1691.75 (10) Å^3^	Block, colourless
*Z* = 2	0.33 × 0.06 × 0.04 mm

### (S)-8-{[(tert-Butyldimethylsilyl)oxy]methyl}-1-[(2,2,4,6,7-pentamethyl-2,3-dihydrobenzofuran-5-yl)sulfonyl]-1,3,4,6,7,8-hexahydro-2H-pyrimido[1,2-a]pyrimidin-1-ium trifluoromethanesulfonate (1) Data collection

**Table:**

Agilent SuperNova, Dual, Cu at zero, Atlas diffractometer	5350 reflections with *I* > 2σ(*I*)
ω scans	*R*_int_ = 0.027
Absorption correction: gaussian (CrysAlisPro; Agilent,2014)	θ_max_ = 73.8°, θ_min_ = 4.5°
*T*_min_ = 0.943, *T*_max_ = 0.988	*h* = −10→10
10136 measured reflections	*k* = −17→17
5593 independent reflections	*l* = −16→16

### (S)-8-{[(tert-Butyldimethylsilyl)oxy]methyl}-1-[(2,2,4,6,7-pentamethyl-2,3-dihydrobenzofuran-5-yl)sulfonyl]-1,3,4,6,7,8-hexahydro-2H-pyrimido[1,2-a]pyrimidin-1-ium trifluoromethanesulfonate (1) Refinement

**Table:**

Refinement on *F*^2^	Hydrogen site location: inferred from neighbouring sites
Least-squares matrix: full	H-atom parameters constrained
*R*[*F*^2^ > 2σ(*F*^2^)] = 0.037	*w* = 1/[σ^2^(*F*_o_^2^) + (0.0537*P*)^2^ + 0.493*P*] where *P* = (*F*_o_^2^ + 2*F*_c_^2^)/3
*wR*(*F*^2^) = 0.097	(Δ/σ)_max_ < 0.001
*S* = 1.02	Δρ_max_ = 0.63 e Å^−^^3^
5593 reflections	Δρ_min_ = −0.47 e Å^−^^3^
408 parameters	Absolute structure: Refined as an inversion twin
1 restraint	Absolute structure parameter: 0.39 (2)
Primary atom site location: structure-invariant direct methods	

### (S)-8-{[(tert-Butyldimethylsilyl)oxy]methyl}-1-[(2,2,4,6,7-pentamethyl-2,3-dihydrobenzofuran-5-yl)sulfonyl]-1,3,4,6,7,8-hexahydro-2H-pyrimido[1,2-a]pyrimidin-1-ium trifluoromethanesulfonate (1) Special details

**Table:**

Geometry. All esds (except the esd in the dihedral angle between two l.s. planes) are estimated using the full covariance matrix. The cell esds are taken into account individually in the estimation of esds in distances, angles and torsion angles; correlations between esds in cell parameters are only used when they are defined by crystal symmetry. An approximate (isotropic) treatment of cell esds is used for estimating esds involving l.s. planes.
Refinement. Refined as a 2-component inversion twin.

### (S)-8-{[(tert-Butyldimethylsilyl)oxy]methyl}-1-[(2,2,4,6,7-pentamethyl-2,3-dihydrobenzofuran-5-yl)sulfonyl]-1,3,4,6,7,8-hexahydro-2H-pyrimido[1,2-a]pyrimidin-1-ium trifluoromethanesulfonate (1) Fractional atomic coordinates and isotropic or equivalent isotropic displacement parameters (Å^2^)

**Table:**

	*x*	*y*	*z*	*U*_iso_*/*U*_eq_	
C1	0.6033 (3)	0.0629 (2)	0.0845 (2)	0.0240 (6)	
C2	0.3643 (4)	0.1575 (2)	0.0936 (2)	0.0284 (7)	
H2	0.373994	0.218073	0.059559	0.034*	
C3	0.2893 (4)	0.0884 (3)	0.0195 (3)	0.0332 (7)	
H3A	0.262817	0.030939	0.053364	0.040*	
H3B	0.191646	0.114320	−0.014690	0.040*	
C4	0.4044 (4)	0.0677 (3)	−0.0534 (3)	0.0350 (7)	
H4A	0.357854	0.022887	−0.102633	0.042*	
H4B	0.428321	0.125130	−0.088146	0.042*	
C5	0.6408 (5)	−0.0298 (3)	−0.0615 (3)	0.0458 (10)	
H5A	0.689976	0.008522	−0.109703	0.055*	
H5B	0.569935	−0.074534	−0.098413	0.055*	
C6	0.7667 (5)	−0.0816 (3)	0.0015 (4)	0.0502 (10)	
H6A	0.718912	−0.129145	0.040960	0.060*	
H6B	0.838164	−0.112741	−0.040288	0.060*	
C7	0.8560 (4)	−0.0128 (3)	0.0679 (3)	0.0403 (8)	
H7A	0.939829	−0.044524	0.110425	0.048*	
H7B	0.905340	0.033850	0.028138	0.048*	
C8	0.2761 (4)	0.1728 (3)	0.1813 (3)	0.0333 (7)	
H8A	0.170991	0.198660	0.160565	0.040*	
H8B	0.262903	0.113620	0.215726	0.040*	
C9	0.3773 (6)	0.1475 (3)	0.4330 (3)	0.0469 (9)	
H9A	0.298018	0.101375	0.410458	0.070*	
H9B	0.371689	0.160347	0.502847	0.070*	
H9C	0.481673	0.123899	0.423649	0.070*	
C10	0.1355 (5)	0.2941 (3)	0.3702 (4)	0.0518 (11)	
H10A	0.112109	0.348206	0.328246	0.078*	
H10B	0.123141	0.310132	0.438453	0.078*	
H10C	0.063191	0.243892	0.348736	0.078*	
C11	0.4901 (4)	0.3470 (3)	0.3994 (3)	0.0329 (7)	
C12	0.4700 (7)	0.3821 (4)	0.5020 (4)	0.0589 (12)	
H12A	0.484443	0.330792	0.548814	0.088*	
H12B	0.364584	0.407852	0.502918	0.088*	
H12C	0.548150	0.430070	0.520430	0.088*	
C13	0.4753 (8)	0.4294 (4)	0.3298 (4)	0.0747 (18)	
H13A	0.369261	0.455009	0.327393	0.112*	
H13B	0.495358	0.409358	0.263920	0.112*	
H13C	0.551844	0.476737	0.353329	0.112*	
C14	0.6544 (6)	0.3066 (4)	0.4006 (5)	0.0684 (15)	
H14A	0.732249	0.354354	0.420425	0.103*	
H14B	0.670084	0.284193	0.334761	0.103*	
H14C	0.666527	0.255118	0.447235	0.103*	
C15	0.8052 (4)	−0.0894 (2)	0.2787 (2)	0.0250 (6)	
C16	0.9498 (3)	−0.1338 (2)	0.3094 (2)	0.0266 (6)	
C17	0.9419 (4)	−0.2285 (3)	0.3241 (2)	0.0299 (7)	
C18	0.8017 (4)	−0.2756 (2)	0.3106 (3)	0.0293 (7)	
C19	0.6591 (4)	−0.2337 (2)	0.2825 (2)	0.0274 (6)	
C20	0.6615 (3)	−0.1383 (2)	0.2653 (2)	0.0251 (6)	
C21	1.0691 (4)	−0.2967 (3)	0.3563 (4)	0.0446 (9)	
H21A	1.111684	−0.285926	0.425402	0.053*	
H21B	1.155647	−0.293256	0.314050	0.053*	
C22	0.9847 (4)	−0.3900 (3)	0.3449 (3)	0.0383 (8)	
C23	1.1083 (4)	−0.0882 (3)	0.3296 (3)	0.0348 (7)	
H23A	1.184820	−0.133406	0.358473	0.052*	
H23B	1.101159	−0.036815	0.375526	0.052*	
H23C	1.141792	−0.064792	0.268038	0.052*	
C24	0.5107 (4)	−0.2903 (3)	0.2726 (3)	0.0340 (7)	
H24A	0.536982	−0.355774	0.282412	0.051*	
H24B	0.455493	−0.281562	0.206879	0.051*	
H24C	0.443114	−0.270473	0.322062	0.051*	
C25	0.5054 (4)	−0.0929 (3)	0.2334 (3)	0.0334 (7)	
H25A	0.481814	−0.098784	0.162068	0.050*	
H25B	0.510525	−0.027348	0.251311	0.050*	
H25C	0.422930	−0.123127	0.266095	0.050*	
C26	1.0218 (5)	−0.4408 (3)	0.2530 (3)	0.0518 (10)	
H26A	0.958062	−0.497013	0.244839	0.078*	
H26B	1.133183	−0.457475	0.259351	0.078*	
H26C	0.998221	−0.400819	0.195675	0.078*	
C27	1.0088 (5)	−0.4488 (3)	0.4357 (3)	0.0486 (10)	
H27A	0.981648	−0.413149	0.492346	0.073*	
H27B	1.118750	−0.468025	0.446673	0.073*	
H27C	0.941545	−0.503578	0.427334	0.073*	
C28	0.8065 (5)	0.3074 (3)	0.0768 (4)	0.0524 (11)	
N1	0.5220 (3)	0.12491 (19)	0.1289 (2)	0.0263 (5)	
H1	0.566303	0.148499	0.184230	0.032*	
N2	0.5494 (3)	0.0295 (2)	−0.00255 (19)	0.0291 (5)	
N3	0.7461 (3)	0.0332 (2)	0.1294 (2)	0.0300 (6)	
O1	0.3655 (3)	0.23600 (18)	0.24468 (18)	0.0356 (6)	
O2	0.6885 (3)	0.07815 (18)	0.30011 (17)	0.0330 (5)	
O3	0.9590 (3)	0.06521 (18)	0.2604 (2)	0.0377 (6)	
O4	0.8158 (3)	−0.36761 (18)	0.3294 (2)	0.0407 (6)	
O5	0.8248 (4)	0.3436 (3)	−0.1041 (3)	0.0778 (13)	
O6	0.7409 (5)	0.1900 (3)	−0.0587 (3)	0.0731 (11)	
O7	1.0094 (4)	0.2368 (3)	−0.0229 (3)	0.0648 (10)	
Si1	0.33991 (10)	0.25617 (7)	0.36096 (7)	0.0305 (2)	
S1	0.80381 (8)	0.03078 (5)	0.25091 (5)	0.02638 (16)	
S2	0.84970 (11)	0.26548 (7)	−0.04097 (7)	0.0423 (2)	
F1	0.6552 (4)	0.3303 (3)	0.0731 (4)	0.1025 (16)	
F2	0.8335 (4)	0.2457 (2)	0.1485 (2)	0.0695 (8)	
F3	0.8890 (5)	0.3835 (2)	0.1023 (3)	0.0914 (13)	

### (S)-8-{[(tert-Butyldimethylsilyl)oxy]methyl}-1-[(2,2,4,6,7-pentamethyl-2,3-dihydrobenzofuran-5-yl)sulfonyl]-1,3,4,6,7,8-hexahydro-2H-pyrimido[1,2-a]pyrimidin-1-ium trifluoromethanesulfonate (1) Atomic displacement parameters (Å^2^)

**Table:**

	*U*^11^	*U*^22^	*U*^33^	*U*^12^	*U*^13^	*U*^23^
C1	0.0235 (14)	0.0214 (14)	0.0286 (14)	−0.0013 (11)	0.0101 (11)	0.0019 (11)
C2	0.0252 (15)	0.0261 (16)	0.0332 (16)	0.0028 (12)	−0.0006 (12)	0.0008 (13)
C3	0.0277 (16)	0.0345 (18)	0.0357 (17)	0.0004 (14)	−0.0040 (13)	−0.0014 (14)
C4	0.0370 (18)	0.0371 (18)	0.0301 (16)	−0.0033 (15)	−0.0007 (13)	−0.0007 (14)
C5	0.041 (2)	0.059 (3)	0.039 (2)	0.0010 (19)	0.0118 (16)	−0.0197 (18)
C6	0.050 (2)	0.044 (2)	0.059 (3)	0.0054 (19)	0.022 (2)	−0.012 (2)
C7	0.0326 (18)	0.053 (2)	0.0379 (18)	0.0119 (17)	0.0142 (14)	0.0007 (17)
C8	0.0246 (15)	0.0392 (18)	0.0353 (17)	0.0030 (14)	−0.0002 (13)	−0.0051 (15)
C9	0.057 (2)	0.0314 (19)	0.053 (2)	0.0008 (17)	0.0131 (19)	0.0044 (17)
C10	0.038 (2)	0.052 (3)	0.067 (3)	0.0096 (19)	0.0119 (19)	−0.014 (2)
C11	0.0388 (18)	0.0284 (17)	0.0310 (16)	0.0004 (14)	0.0012 (13)	0.0007 (14)
C12	0.082 (3)	0.052 (3)	0.044 (2)	−0.016 (2)	0.014 (2)	−0.014 (2)
C13	0.105 (5)	0.052 (3)	0.060 (3)	−0.026 (3)	−0.026 (3)	0.024 (2)
C14	0.040 (2)	0.064 (3)	0.100 (4)	−0.004 (2)	−0.001 (2)	−0.035 (3)
C15	0.0213 (14)	0.0279 (15)	0.0264 (14)	0.0009 (12)	0.0058 (11)	0.0007 (12)
C16	0.0192 (14)	0.0317 (17)	0.0291 (15)	0.0006 (12)	0.0039 (11)	−0.0043 (13)
C17	0.0204 (14)	0.0327 (17)	0.0367 (16)	0.0035 (13)	0.0046 (12)	−0.0001 (14)
C18	0.0268 (16)	0.0246 (15)	0.0370 (17)	0.0025 (12)	0.0063 (13)	0.0020 (13)
C19	0.0215 (14)	0.0315 (16)	0.0298 (14)	−0.0033 (13)	0.0056 (11)	−0.0024 (13)
C20	0.0164 (13)	0.0314 (16)	0.0281 (14)	0.0012 (12)	0.0049 (11)	0.0000 (12)
C21	0.0267 (17)	0.0324 (19)	0.074 (3)	0.0048 (15)	0.0039 (17)	0.0027 (18)
C22	0.0313 (17)	0.0317 (18)	0.053 (2)	0.0074 (14)	0.0080 (15)	0.0009 (16)
C23	0.0190 (15)	0.0342 (18)	0.050 (2)	−0.0001 (13)	−0.0020 (13)	−0.0014 (15)
C24	0.0251 (16)	0.0357 (19)	0.0411 (18)	−0.0060 (14)	0.0034 (13)	−0.0011 (15)
C25	0.0152 (13)	0.0419 (19)	0.0429 (19)	−0.0020 (13)	0.0028 (12)	0.0093 (16)
C26	0.047 (2)	0.054 (3)	0.056 (2)	0.0102 (19)	0.0099 (19)	−0.004 (2)
C27	0.045 (2)	0.049 (3)	0.052 (2)	0.0091 (18)	0.0093 (17)	0.0053 (19)
C28	0.050 (2)	0.0266 (18)	0.085 (3)	−0.0061 (18)	0.029 (2)	−0.004 (2)
N1	0.0231 (13)	0.0224 (13)	0.0328 (13)	0.0037 (10)	0.0005 (10)	−0.0051 (10)
N2	0.0317 (13)	0.0289 (13)	0.0275 (12)	−0.0013 (12)	0.0065 (10)	−0.0021 (12)
N3	0.0235 (12)	0.0345 (14)	0.0334 (13)	0.0062 (12)	0.0087 (10)	0.0008 (13)
O1	0.0331 (12)	0.0372 (14)	0.0368 (13)	−0.0004 (10)	0.0046 (10)	−0.0081 (11)
O2	0.0307 (12)	0.0371 (13)	0.0309 (12)	0.0100 (10)	0.0022 (9)	−0.0083 (10)
O3	0.0244 (12)	0.0284 (12)	0.0587 (16)	−0.0032 (9)	−0.0027 (10)	0.0003 (11)
O4	0.0294 (13)	0.0270 (12)	0.0664 (18)	0.0031 (10)	0.0083 (12)	0.0015 (12)
O5	0.0482 (19)	0.088 (3)	0.094 (3)	−0.0116 (18)	−0.0053 (18)	0.059 (2)
O6	0.092 (3)	0.073 (2)	0.0518 (19)	−0.040 (2)	−0.0033 (18)	0.0070 (18)
O7	0.0530 (19)	0.069 (2)	0.073 (2)	0.0229 (17)	0.0087 (16)	−0.0075 (19)
Si1	0.0291 (4)	0.0282 (4)	0.0349 (4)	0.0042 (4)	0.0066 (3)	−0.0028 (4)
S1	0.0200 (3)	0.0256 (4)	0.0333 (4)	0.0017 (3)	0.0019 (3)	−0.0027 (3)
S2	0.0404 (5)	0.0408 (5)	0.0434 (5)	−0.0072 (4)	−0.0063 (3)	0.0111 (4)
F1	0.058 (2)	0.075 (2)	0.185 (5)	0.0069 (16)	0.063 (3)	0.003 (3)
F2	0.089 (2)	0.074 (2)	0.0463 (14)	−0.0234 (17)	0.0077 (13)	−0.0024 (14)
F3	0.106 (3)	0.0572 (19)	0.120 (3)	−0.0416 (19)	0.052 (2)	−0.047 (2)

### (S)-8-{[(tert-Butyldimethylsilyl)oxy]methyl}-1-[(2,2,4,6,7-pentamethyl-2,3-dihydrobenzofuran-5-yl)sulfonyl]-1,3,4,6,7,8-hexahydro-2H-pyrimido[1,2-a]pyrimidin-1-ium trifluoromethanesulfonate (1) Geometric parameters (Å, º)

**Table:**

C1—N1	1.323 (4)	C14—H14C	0.9800
C1—N2	1.324 (4)	C15—C20	1.416 (4)
C1—N3	1.380 (4)	C15—C16	1.421 (4)
C2—N1	1.466 (4)	C15—S1	1.781 (3)
C2—C8	1.503 (5)	C16—C17	1.388 (5)
C2—C3	1.519 (5)	C16—C23	1.511 (4)
C2—H2	1.0000	C17—C18	1.378 (5)
C3—C4	1.506 (5)	C17—C21	1.503 (5)
C3—H3A	0.9900	C18—O4	1.361 (4)
C3—H3B	0.9900	C18—C19	1.383 (5)
C4—N2	1.468 (5)	C19—C20	1.402 (5)
C4—H4A	0.9900	C19—C24	1.508 (4)
C4—H4B	0.9900	C20—C25	1.514 (4)
C5—N2	1.462 (4)	C21—C22	1.533 (6)
C5—C6	1.510 (7)	C21—H21A	0.9900
C5—H5A	0.9900	C21—H21B	0.9900
C5—H5B	0.9900	C22—O4	1.478 (4)
C6—C7	1.503 (6)	C22—C27	1.504 (6)
C6—H6A	0.9900	C22—C26	1.520 (6)
C6—H6B	0.9900	C23—H23A	0.9800
C7—N3	1.486 (4)	C23—H23B	0.9800
C7—H7A	0.9900	C23—H23C	0.9800
C7—H7B	0.9900	C24—H24A	0.9800
C8—O1	1.428 (4)	C24—H24B	0.9800
C8—H8A	0.9900	C24—H24C	0.9800
C8—H8B	0.9900	C25—H25A	0.9800
C9—Si1	1.867 (4)	C25—H25B	0.9800
C9—H9A	0.9800	C25—H25C	0.9800
C9—H9B	0.9800	C26—H26A	0.9800
C9—H9C	0.9800	C26—H26B	0.9800
C10—Si1	1.854 (4)	C26—H26C	0.9800
C10—H10A	0.9800	C27—H27A	0.9800
C10—H10B	0.9800	C27—H27B	0.9800
C10—H10C	0.9800	C27—H27C	0.9800
C11—C12	1.521 (6)	C28—F2	1.330 (6)
C11—C13	1.523 (6)	C28—F1	1.335 (6)
C11—C14	1.525 (6)	C28—F3	1.336 (5)
C11—Si1	1.878 (4)	C28—S2	1.798 (5)
C12—H12A	0.9800	N1—H1	0.8800
C12—H12B	0.9800	N3—S1	1.686 (3)
C12—H12C	0.9800	O1—Si1	1.656 (3)
C13—H13A	0.9800	O2—S1	1.429 (2)
C13—H13B	0.9800	O3—S1	1.415 (2)
C13—H13C	0.9800	O5—S2	1.426 (4)
C14—H14A	0.9800	O6—S2	1.441 (4)
C14—H14B	0.9800	O7—S2	1.428 (4)
			
N1—C1—N2	120.7 (3)	C18—C17—C21	108.1 (3)
N1—C1—N3	119.5 (3)	C16—C17—C21	130.3 (3)
N2—C1—N3	119.8 (3)	O4—C18—C17	113.6 (3)
N1—C2—C8	107.9 (3)	O4—C18—C19	122.7 (3)
N1—C2—C3	108.6 (3)	C17—C18—C19	123.7 (3)
C8—C2—C3	114.7 (3)	C18—C19—C20	116.7 (3)
N1—C2—H2	108.5	C18—C19—C24	120.0 (3)
C8—C2—H2	108.5	C20—C19—C24	123.2 (3)
C3—C2—H2	108.5	C19—C20—C15	119.9 (3)
C4—C3—C2	108.2 (3)	C19—C20—C25	116.6 (3)
C4—C3—H3A	110.1	C15—C20—C25	123.5 (3)
C2—C3—H3A	110.1	C17—C21—C22	103.2 (3)
C4—C3—H3B	110.1	C17—C21—H21A	111.1
C2—C3—H3B	110.1	C22—C21—H21A	111.1
H3A—C3—H3B	108.4	C17—C21—H21B	111.1
N2—C4—C3	110.0 (3)	C22—C21—H21B	111.1
N2—C4—H4A	109.7	H21A—C21—H21B	109.1
C3—C4—H4A	109.7	O4—C22—C27	106.8 (3)
N2—C4—H4B	109.7	O4—C22—C26	106.0 (3)
C3—C4—H4B	109.7	C27—C22—C26	112.8 (3)
H4A—C4—H4B	108.2	O4—C22—C21	105.4 (3)
N2—C5—C6	111.7 (3)	C27—C22—C21	113.1 (4)
N2—C5—H5A	109.3	C26—C22—C21	111.9 (4)
C6—C5—H5A	109.3	C16—C23—H23A	109.5
N2—C5—H5B	109.3	C16—C23—H23B	109.5
C6—C5—H5B	109.3	H23A—C23—H23B	109.5
H5A—C5—H5B	107.9	C16—C23—H23C	109.5
C7—C6—C5	107.7 (4)	H23A—C23—H23C	109.5
C7—C6—H6A	110.2	H23B—C23—H23C	109.5
C5—C6—H6A	110.2	C19—C24—H24A	109.5
C7—C6—H6B	110.2	C19—C24—H24B	109.5
C5—C6—H6B	110.2	H24A—C24—H24B	109.5
H6A—C6—H6B	108.5	C19—C24—H24C	109.5
N3—C7—C6	109.1 (3)	H24A—C24—H24C	109.5
N3—C7—H7A	109.9	H24B—C24—H24C	109.5
C6—C7—H7A	109.9	C20—C25—H25A	109.5
N3—C7—H7B	109.9	C20—C25—H25B	109.5
C6—C7—H7B	109.9	H25A—C25—H25B	109.5
H7A—C7—H7B	108.3	C20—C25—H25C	109.5
O1—C8—C2	107.3 (3)	H25A—C25—H25C	109.5
O1—C8—H8A	110.3	H25B—C25—H25C	109.5
C2—C8—H8A	110.3	C22—C26—H26A	109.5
O1—C8—H8B	110.3	C22—C26—H26B	109.5
C2—C8—H8B	110.3	H26A—C26—H26B	109.5
H8A—C8—H8B	108.5	C22—C26—H26C	109.5
Si1—C9—H9A	109.5	H26A—C26—H26C	109.5
Si1—C9—H9B	109.5	H26B—C26—H26C	109.5
H9A—C9—H9B	109.5	C22—C27—H27A	109.5
Si1—C9—H9C	109.5	C22—C27—H27B	109.5
H9A—C9—H9C	109.5	H27A—C27—H27B	109.5
H9B—C9—H9C	109.5	C22—C27—H27C	109.5
Si1—C10—H10A	109.5	H27A—C27—H27C	109.5
Si1—C10—H10B	109.5	H27B—C27—H27C	109.5
H10A—C10—H10B	109.5	F2—C28—F1	106.7 (4)
Si1—C10—H10C	109.5	F2—C28—F3	108.3 (5)
H10A—C10—H10C	109.5	F1—C28—F3	106.9 (4)
H10B—C10—H10C	109.5	F2—C28—S2	113.6 (3)
C12—C11—C13	107.9 (4)	F1—C28—S2	110.0 (4)
C12—C11—C14	108.2 (4)	F3—C28—S2	111.1 (3)
C13—C11—C14	108.9 (5)	C1—N1—C2	125.4 (3)
C12—C11—Si1	110.6 (3)	C1—N1—H1	117.3
C13—C11—Si1	111.3 (3)	C2—N1—H1	117.3
C14—C11—Si1	109.9 (3)	C1—N2—C5	123.7 (3)
C11—C12—H12A	109.5	C1—N2—C4	119.1 (3)
C11—C12—H12B	109.5	C5—N2—C4	115.9 (3)
H12A—C12—H12B	109.5	C1—N3—C7	118.3 (3)
C11—C12—H12C	109.5	C1—N3—S1	126.9 (2)
H12A—C12—H12C	109.5	C7—N3—S1	114.3 (2)
H12B—C12—H12C	109.5	C8—O1—Si1	125.5 (2)
C11—C13—H13A	109.5	C18—O4—C22	108.0 (3)
C11—C13—H13B	109.5	O1—Si1—C10	109.86 (19)
H13A—C13—H13B	109.5	O1—Si1—C9	109.10 (17)
C11—C13—H13C	109.5	C10—Si1—C9	108.9 (2)
H13A—C13—H13C	109.5	O1—Si1—C11	103.67 (15)
H13B—C13—H13C	109.5	C10—Si1—C11	113.55 (19)
C11—C14—H14A	109.5	C9—Si1—C11	111.59 (19)
C11—C14—H14B	109.5	O3—S1—O2	118.63 (16)
H14A—C14—H14B	109.5	O3—S1—N3	104.99 (15)
C11—C14—H14C	109.5	O2—S1—N3	108.02 (14)
H14A—C14—H14C	109.5	O3—S1—C15	109.86 (15)
H14B—C14—H14C	109.5	O2—S1—C15	110.96 (15)
C20—C15—C16	122.2 (3)	N3—S1—C15	103.06 (15)
C20—C15—S1	118.3 (2)	O5—S2—O7	114.6 (2)
C16—C15—S1	119.4 (2)	O5—S2—O6	116.5 (2)
C17—C16—C15	115.8 (3)	O7—S2—O6	113.6 (3)
C17—C16—C23	117.5 (3)	O5—S2—C28	104.1 (3)
C15—C16—C23	126.7 (3)	O7—S2—C28	103.2 (2)
C18—C17—C16	121.6 (3)	O6—S2—C28	102.5 (2)

### (S)-8-{[(tert-Butyldimethylsilyl)oxy]methyl}-1-[(2,2,4,6,7-pentamethyl-2,3-dihydrobenzofuran-5-yl)sulfonyl]-1,3,4,6,7,8-hexahydro-2H-pyrimido[1,2-a]pyrimidin-1-ium trifluoromethanesulfonate (1) Hydrogen-bond geometry (Å, º)

**Table:**

*D*—H···*A*	*D*—H	H···*A*	*D*···*A*	*D*—H···*A*
N1—H1···O1	0.88	2.36	2.712 (4)	104
N1—H1···O2	0.88	2.07	2.700 (4)	127
C3—H3*B*···O7^i^	0.99	2.36	3.229 (5)	146
C5—H5*B*···F1^ii^	0.99	2.43	3.239 (6)	139
C24—H24*A*···O4	0.98	2.42	2.877 (4)	108
C25—H25*B*···O2	0.98	2.21	3.022 (5)	139
C7—H7*A*···O5^iii^	0.99	2.60	3.433 (5)	142
C24—H24*B*···O6^ii^	0.98	2.53	3.462 (5)	159

Symmetry codes: (i) *x*−1, *y*, *z*; (ii) −*x*+1, *y*−1/2, −*z*; (iii) −*x*+2, *y*−1/2, −*z*.

### (S)-8-(Iodomethyl)-1-4-(methylbenzenesulfonyl)-1,3,4,6,7,8-hexahydro-2H-pyrimido[1,2-a]pyrimidin-1-ium iodide (2) Crystal data

**Table:**

C_15_H_21_IN_3_O_2_S^+^·I^−^	*D*_x_ = 1.974 Mg m^−^^3^
*M**_r_* = 561.21	Mo *K*α radiation, λ = 0.71073 Å
Orthorhombic, *P*2_1_2_1_2_1_	Cell parameters from 3858 reflections
*a* = 6.6117 (2) Å	θ = 3.0–27.4°
*b* = 10.1482 (2) Å	µ = 3.45 mm^−^^1^
*c* = 28.1444 (9) Å	*T* = 150 K
*V* = 1888.40 (9) Å^3^	Needle, colourless
*Z* = 4	0.41 × 0.20 × 0.20 mm
*F*(000) = 1080	

### (S)-8-(Iodomethyl)-1-4-(methylbenzenesulfonyl)-1,3,4,6,7,8-hexahydro-2H-pyrimido[1,2-a]pyrimidin-1-ium iodide (2) Data collection

**Table:**

Nonius KappaCCD diffractometer	3858 reflections with *I* > 2σ(*I*)
CCD scans	*R*_int_ = 0.030
Absorption correction: empirical (using intensity measurements) Denzo/Scalepack	θ_max_ = 27.4°, θ_min_ = 2.1°
*T*_min_ = 0.339, *T*_max_ = 0.545	*h* = −8→8
4087 measured reflections	*k* = −13→13
4087 independent reflections	*l* = −36→36

### (S)-8-(Iodomethyl)-1-4-(methylbenzenesulfonyl)-1,3,4,6,7,8-hexahydro-2H-pyrimido[1,2-a]pyrimidin-1-ium iodide (2) Refinement

**Table:**

Refinement on *F*^2^	H-atom parameters constrained
Least-squares matrix: full	*w* = 1/[σ^2^(*F*_o_^2^) + (0.0251*P*)^2^ + 5.6778*P*] where *P* = (*F*_o_^2^ + 2*F*_c_^2^)/3
*R*[*F*^2^ > 2σ(*F*^2^)] = 0.031	(Δ/σ)_max_ = 0.001
*wR*(*F*^2^) = 0.072	Δρ_max_ = 0.77 e Å^−^^3^
*S* = 1.04	Δρ_min_ = −0.79 e Å^−^^3^
4087 reflections	Extinction correction: SHELXL-2018/1 (Sheldrick 2018), Fc^*^=kFc[1+0.001xFc^2^λ^3^/sin(2θ)]^-1/4^
207 parameters	Extinction coefficient: 0.0068 (4)
0 restraints	Absolute structure: Flack x determined using 1475 quotients [(I+)-(I-)]/[(I+)+(I-)] (Parsons, Flack and Wagner, Acta Cryst. B69 (2013) 249-259).
Hydrogen site location: inferred from neighbouring sites	Absolute structure parameter: −0.002 (19)

### (S)-8-(Iodomethyl)-1-4-(methylbenzenesulfonyl)-1,3,4,6,7,8-hexahydro-2H-pyrimido[1,2-a]pyrimidin-1-ium iodide (2) Special details

**Table:**

Geometry. All esds (except the esd in the dihedral angle between two l.s. planes) are estimated using the full covariance matrix. The cell esds are taken into account individually in the estimation of esds in distances, angles and torsion angles; correlations between esds in cell parameters are only used when they are defined by crystal symmetry. An approximate (isotropic) treatment of cell esds is used for estimating esds involving l.s. planes.

### (S)-8-(Iodomethyl)-1-4-(methylbenzenesulfonyl)-1,3,4,6,7,8-hexahydro-2H-pyrimido[1,2-a]pyrimidin-1-ium iodide (2) Fractional atomic coordinates and isotropic or equivalent isotropic displacement parameters (Å^2^)

**Table:**

	*x*	*y*	*z*	*U*_iso_*/*U*_eq_	
C1	0.6440 (9)	0.2979 (6)	0.3545 (2)	0.0176 (12)	
C2	0.8412 (10)	0.4145 (6)	0.2919 (2)	0.0200 (12)	
H2	0.949093	0.357300	0.277960	0.024*	
C3	0.6515 (10)	0.4008 (6)	0.2620 (2)	0.0224 (13)	
H3A	0.557522	0.474061	0.269212	0.027*	
H3B	0.687244	0.405578	0.227891	0.027*	
C4	0.5492 (11)	0.2707 (7)	0.2723 (2)	0.0263 (15)	
H4A	0.421253	0.264838	0.254181	0.032*	
H4B	0.637911	0.197383	0.262082	0.032*	
C5	0.3238 (11)	0.1838 (6)	0.3364 (2)	0.0272 (13)	
H5A	0.340605	0.090934	0.326324	0.033*	
H5B	0.206408	0.220687	0.319050	0.033*	
C6	0.2819 (9)	0.1881 (7)	0.3890 (2)	0.0245 (14)	
H6A	0.178509	0.121590	0.397449	0.029*	
H6B	0.230543	0.276178	0.398078	0.029*	
C7	0.4773 (10)	0.1594 (6)	0.4148 (2)	0.0231 (14)	
H7A	0.453776	0.158156	0.449487	0.028*	
H7B	0.530122	0.072116	0.405091	0.028*	
C8	0.9149 (9)	0.5559 (7)	0.2918 (2)	0.0227 (13)	
H8A	0.806441	0.613563	0.304233	0.027*	
H8B	0.942861	0.582957	0.258602	0.027*	
C9	0.5690 (9)	0.5036 (6)	0.4450 (2)	0.0187 (13)	
C10	0.3777 (9)	0.4989 (6)	0.4664 (2)	0.0195 (13)	
H10	0.329194	0.420139	0.480654	0.023*	
C11	0.2610 (10)	0.6127 (7)	0.4662 (2)	0.0241 (14)	
H11	0.132677	0.611967	0.481372	0.029*	
C12	0.3273 (11)	0.7277 (6)	0.4442 (2)	0.0219 (12)	
C13	0.5197 (10)	0.7297 (7)	0.4230 (3)	0.0259 (15)	
H13	0.567125	0.807997	0.408247	0.031*	
C14	0.6406 (10)	0.6182 (6)	0.4236 (2)	0.0223 (14)	
H14	0.771147	0.619926	0.409492	0.027*	
C15	0.1948 (13)	0.8482 (7)	0.4420 (3)	0.0351 (17)	
H15A	0.068847	0.831523	0.459393	0.053*	
H15B	0.163584	0.868658	0.408810	0.053*	
H15C	0.265650	0.922976	0.456504	0.053*	
I1	1.18349 (7)	0.58374 (4)	0.33364 (2)	0.02686 (13)	
I2	0.17276 (7)	0.45181 (4)	0.17516 (2)	0.02671 (13)	
N1	0.801213	0.370167	0.340930	0.0209 (10)	
H1	0.888533	0.393510	0.362975	0.025*	
N2	0.5063 (7)	0.2585 (5)	0.3237 (2)	0.0218 (11)	
N3	0.6254 (8)	0.2639 (5)	0.40248 (18)	0.0176 (11)	
O1	0.9203 (6)	0.3955 (5)	0.43363 (16)	0.0234 (10)	
O2	0.6798 (7)	0.2891 (4)	0.48886 (14)	0.0233 (9)	
S1	0.7169 (2)	0.36092 (16)	0.44604 (6)	0.0184 (3)	

### (S)-8-(Iodomethyl)-1-4-(methylbenzenesulfonyl)-1,3,4,6,7,8-hexahydro-2H-pyrimido[1,2-a]pyrimidin-1-ium iodide (2) Atomic displacement parameters (Å^2^)

**Table:**

	*U*^11^	*U*^22^	*U*^33^	*U*^12^	*U*^13^	*U*^23^
C1	0.021 (3)	0.015 (3)	0.017 (3)	0.003 (2)	0.000 (2)	−0.002 (2)
C2	0.023 (3)	0.020 (3)	0.017 (3)	−0.001 (3)	0.001 (2)	0.002 (2)
C3	0.030 (3)	0.024 (3)	0.014 (3)	0.002 (3)	−0.003 (3)	0.002 (2)
C4	0.033 (4)	0.033 (4)	0.013 (3)	0.000 (3)	−0.001 (3)	0.000 (3)
C5	0.024 (3)	0.025 (3)	0.032 (4)	−0.004 (3)	−0.002 (3)	−0.001 (3)
C6	0.019 (3)	0.025 (3)	0.029 (4)	−0.004 (3)	0.001 (3)	−0.002 (3)
C7	0.023 (3)	0.021 (3)	0.025 (3)	−0.004 (3)	0.002 (3)	0.000 (3)
C8	0.021 (3)	0.028 (4)	0.020 (3)	0.002 (3)	−0.005 (2)	0.002 (3)
C9	0.017 (3)	0.022 (3)	0.018 (3)	−0.003 (2)	−0.002 (2)	−0.003 (2)
C10	0.021 (3)	0.018 (3)	0.020 (3)	−0.003 (2)	0.000 (2)	0.003 (2)
C11	0.023 (3)	0.023 (3)	0.026 (3)	0.000 (2)	0.001 (2)	−0.004 (3)
C12	0.027 (3)	0.019 (3)	0.020 (3)	0.002 (3)	−0.001 (3)	−0.001 (2)
C13	0.031 (4)	0.022 (3)	0.025 (4)	−0.002 (3)	0.003 (3)	0.001 (3)
C14	0.025 (4)	0.022 (3)	0.020 (3)	−0.008 (3)	0.000 (2)	−0.001 (2)
C15	0.045 (4)	0.029 (4)	0.032 (4)	0.011 (4)	0.001 (4)	−0.002 (3)
I1	0.0253 (2)	0.0316 (2)	0.0236 (2)	−0.00409 (18)	0.00070 (18)	0.00262 (17)
I2	0.0248 (2)	0.0301 (2)	0.0253 (2)	−0.00338 (18)	0.00167 (18)	−0.00130 (17)
N1	0.019 (2)	0.025 (3)	0.019 (2)	0.001 (2)	−0.001 (2)	0.001 (2)
N2	0.020 (3)	0.028 (3)	0.017 (3)	−0.002 (2)	−0.003 (2)	0.001 (2)
N3	0.022 (3)	0.018 (3)	0.013 (2)	−0.002 (2)	0.0020 (19)	0.003 (2)
O1	0.015 (2)	0.036 (3)	0.020 (2)	−0.0010 (19)	0.0012 (17)	−0.002 (2)
O2	0.029 (2)	0.027 (2)	0.0144 (19)	0.002 (2)	−0.001 (2)	0.0046 (17)
S1	0.0193 (8)	0.0210 (7)	0.0149 (7)	0.0010 (6)	−0.0015 (5)	0.0006 (6)

### (S)-8-(Iodomethyl)-1-4-(methylbenzenesulfonyl)-1,3,4,6,7,8-hexahydro-2H-pyrimido[1,2-a]pyrimidin-1-ium iodide (2) Geometric parameters (Å, º)

**Table:**

C1—N2	1.318 (8)	C8—I1	2.150 (6)
C1—N1	1.328 (6)	C8—H8A	0.9900
C1—N3	1.400 (7)	C8—H8B	0.9900
C2—N1	1.475 (6)	C9—C14	1.394 (9)
C2—C8	1.516 (9)	C9—C10	1.402 (8)
C2—C3	1.517 (9)	C9—S1	1.747 (6)
C2—H2	1.0000	C10—C11	1.389 (9)
C3—C4	1.511 (10)	C10—H10	0.9500
C3—H3A	0.9900	C11—C12	1.391 (9)
C3—H3B	0.9900	C11—H11	0.9500
C4—N2	1.479 (8)	C12—C13	1.405 (10)
C4—H4A	0.9900	C12—C15	1.506 (9)
C4—H4B	0.9900	C13—C14	1.386 (10)
C5—N2	1.469 (8)	C13—H13	0.9500
C5—C6	1.507 (9)	C14—H14	0.9500
C5—H5A	0.9900	C15—H15A	0.9800
C5—H5B	0.9900	C15—H15B	0.9800
C6—C7	1.510 (9)	C15—H15C	0.9800
C6—H6A	0.9900	N1—H1	0.8800
C6—H6B	0.9900	N3—S1	1.685 (5)
C7—N3	1.484 (8)	O1—S1	1.433 (4)
C7—H7A	0.9900	O2—S1	1.429 (4)
C7—H7B	0.9900		
			
N2—C1—N1	121.3 (5)	C2—C8—H8B	109.0
N2—C1—N3	119.9 (6)	I1—C8—H8B	109.0
N1—C1—N3	118.8 (5)	H8A—C8—H8B	107.8
N1—C2—C8	110.4 (5)	C14—C9—C10	121.4 (6)
N1—C2—C3	110.1 (5)	C14—C9—S1	120.6 (5)
C8—C2—C3	110.5 (5)	C10—C9—S1	118.0 (5)
N1—C2—H2	108.6	C11—C10—C9	118.1 (6)
C8—C2—H2	108.6	C11—C10—H10	120.9
C3—C2—H2	108.6	C9—C10—H10	120.9
C4—C3—C2	110.1 (5)	C10—C11—C12	121.6 (6)
C4—C3—H3A	109.6	C10—C11—H11	119.2
C2—C3—H3A	109.6	C12—C11—H11	119.2
C4—C3—H3B	109.6	C11—C12—C13	119.1 (6)
C2—C3—H3B	109.6	C11—C12—C15	121.1 (7)
H3A—C3—H3B	108.2	C13—C12—C15	119.8 (6)
N2—C4—C3	110.3 (5)	C14—C13—C12	120.4 (6)
N2—C4—H4A	109.6	C14—C13—H13	119.8
C3—C4—H4A	109.6	C12—C13—H13	119.8
N2—C4—H4B	109.6	C13—C14—C9	119.4 (6)
C3—C4—H4B	109.6	C13—C14—H14	120.3
H4A—C4—H4B	108.1	C9—C14—H14	120.3
N2—C5—C6	112.0 (5)	C12—C15—H15A	109.5
N2—C5—H5A	109.2	C12—C15—H15B	109.5
C6—C5—H5A	109.2	H15A—C15—H15B	109.5
N2—C5—H5B	109.2	C12—C15—H15C	109.5
C6—C5—H5B	109.2	H15A—C15—H15C	109.5
H5A—C5—H5B	107.9	H15B—C15—H15C	109.5
C5—C6—C7	108.0 (5)	C1—N1—C2	125.2 (4)
C5—C6—H6A	110.1	C1—N1—H1	117.4
C7—C6—H6A	110.1	C2—N1—H1	117.4
C5—C6—H6B	110.1	C1—N2—C5	124.4 (6)
C7—C6—H6B	110.1	C1—N2—C4	119.0 (5)
H6A—C6—H6B	108.4	C5—N2—C4	116.0 (5)
N3—C7—C6	108.3 (5)	C1—N3—C7	117.3 (5)
N3—C7—H7A	110.0	C1—N3—S1	121.8 (4)
C6—C7—H7A	110.0	C7—N3—S1	119.0 (4)
N3—C7—H7B	110.0	O2—S1—O1	119.4 (3)
C6—C7—H7B	110.0	O2—S1—N3	104.7 (3)
H7A—C7—H7B	108.4	O1—S1—N3	107.6 (3)
C2—C8—I1	112.8 (4)	O2—S1—C9	109.9 (3)
C2—C8—H8A	109.0	O1—S1—C9	108.6 (3)
I1—C8—H8A	109.0	N3—S1—C9	105.7 (3)

### (S)-8-(Iodomethyl)-1-4-(methylbenzenesulfonyl)-1,3,4,6,7,8-hexahydro-2H-pyrimido[1,2-a]pyrimidin-1-ium iodide (2) Hydrogen-bond geometry (Å, º)

**Table:**

*D*—H···*A*	*D*—H	H···*A*	*D*···*A*	*D*—H···*A*
N1—H1···I1	0.88	2.87	3.3359 (4)	115
N1—H1···O1	0.88	2.00	2.737 (5)	141
C7—H7*A*···O2	0.99	2.29	2.807 (8)	112
C14—H14···O1	0.95	2.57	2.934 (8)	103
C6—H6*B*···O1^i^	0.99	2.58	3.425 (8)	143
C7—H7*A*···O2^ii^	0.99	2.56	3.391 (8)	141
C10—H10···O2^ii^	0.95	2.49	3.441 (7)	174

Symmetry codes: (i) *x*−1, *y*, *z*; (ii) *x*−1/2, −*y*+1/2, −*z*+1.
